# Reversing the Tide: A Case of a Mechanical Aortic Valve Recipient Lost to Follow-up, Education on Rivaroxaban Contraindications, and the Vital Role of Acenocoumarol in Preventing Valve Thrombosis

**DOI:** 10.7759/cureus.57059

**Published:** 2024-03-27

**Authors:** Hasan Kazma, Malak Fakih, Aalaa A Saleh, Yara Tarhini, Malek Mohammed

**Affiliations:** 1 Cardiology, Bahman Hospital, Beirut, LBN; 2 Medicine, Faculty of Medicine, Lebanese University, Beirut, LBN; 3 Cardiology, Faculty of Medicine, Lebanese University, Beirut, LBN; 4 General Medicine, Faculty of Medicine, Lebanese University, Beirut, LBN

**Keywords:** mechanical aortic valve thrombosis, bioprosthetic aortic valve, aortic valve replacement, oral direct anti-coagulant, ribaroxavan, warfarin, acenocoumarol, oral anti-vitamin k, mechanical aortic valve prosthesis, aortic stenosis

## Abstract

Aortic stenosis is the most common heart valve disease, especially among the elderly. Symptomatic aortic valve stenosis is linked to a poor prognosis and a high mortality rate if left untreated. The only effective treatment for severe symptomatic aortic stenosis is aortic valve replacement using either a mechanical or a biological prosthesis. Mechanical valve prostheses, while highly durable, are thrombogenic, necessitating lifelong anticoagulation with oral anti-vitamin K agents, such as acenocoumarol. Conversely, bioprosthetic valves, though less durable, carry a minimal thrombogenic risk and do not require anticoagulation. Currently, there is no proven role for direct-acting oral anticoagulants (DOACs) in patients with mechanical heart valves due to insufficient clinical trial data regarding their safety in this patient population. Herein, we present the case of a 59-year-old female known to have aortic stenosis, who underwent surgical treatment with mechanical aortic valve replacement eight years ago. Post-surgery, acenocoumarol was initiated. However, 18 months prior to presenting at our institution, the patient started taking rivaroxaban (a DOAC) instead of acenocoumarol due to the unavailability of acenocoumarol during the ongoing economic crisis in Lebanon, without consulting her cardiologist. Although she was followed up by her general practitioner and reported having a mechanical valve, her son contradicted this, claiming she had a biological valve. After thorough investigations, including chest X-ray, echocardiography, and fluoroscopy, it was confirmed that the patient indeed had a normally functioning mechanical aortic valve. Immediate corrective measures were taken, starting with IV unfractionated heparin and acenocoumarol, targeting an International Normalized Ratio (INR) between 2.5 and 3, while educating the patient about her condition and the importance of adhering to acenocoumarol therapy.

## Introduction

Aortic valve disease is prevalent, particularly among the elderly, affecting about 25% of those over 65 years of age with aortic sclerosis and 3% over 75 years with severe stenosis. This condition has shifted from its historical association with rheumatic fever to an active inflammatory process with similarities to atherosclerosis [1,2].

Although symptoms are a crucial determinant of outcomes, the later age of onset complicates the distinction of aortic stenosis from other age-related comorbidities. Upon symptom onset, life expectancy drops to approximately 3 years unless aortic valve replacement relieves the mechanical obstruction [2]. Once valve replacement is indicated for symptomatic severe stenosis, the choice of valve type depends on the patient’s age and bleeding risk. Mechanical valves, while durable, require lifelong anticoagulation; conversely, bioprosthetic valves are less durable but do not necessitate anticoagulation [3,4]. The only anticoagulant drugs approved for preventing mechanical valve thrombosis are vitamin K antagonists, such as acenocoumarol and warfarin. Direct-acting oral anticoagulants (DOACs) are not indicated due to the absence of data confirming their safety and efficacy in cases of mechanical valve prostheses [4,5]. In this article, we emphasize the role of vitamin K antagonist use in patients with mechanical heart valve prostheses and underscore the importance of educating primary care physicians and patients about their condition. It is crucial to stress the significance of medication compliance and to highlight that any changes in medication should only be made after consulting with a cardiologist.

## Case presentation

**Figure 1:**
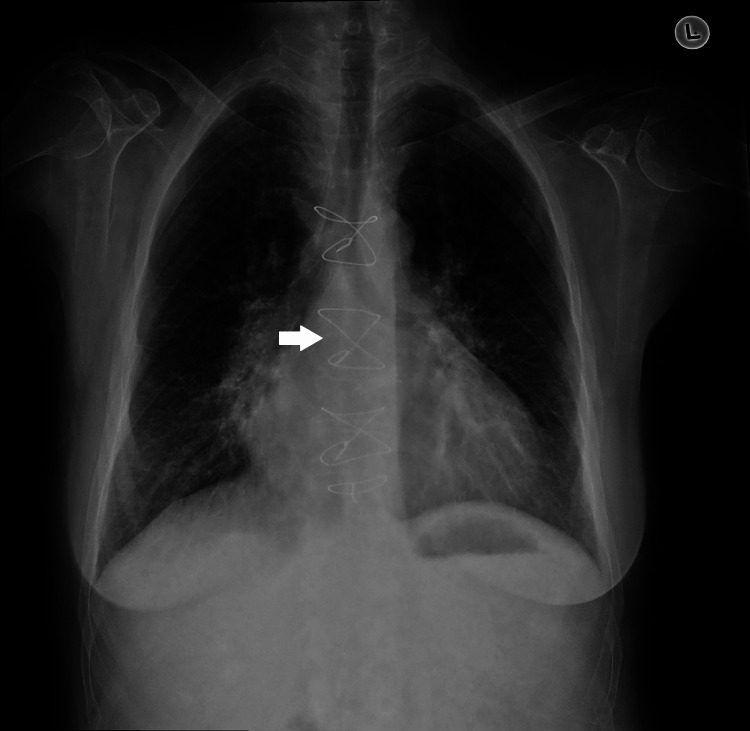
Posteroanterior chest X-ray showing cardiomegaly, clear lungs, hilar prominence, and sternal wires (indicated by a white arrow).

This report involves a case of a 59-year-old female patient, a smoker, with no known food or drug allergies. She has a medical history of hypertension, diabetes mellitus, chronic kidney disease with a last recorded creatinine level of 2.5 mg/dl, chronic obstructive pulmonary disease, heart failure with recurrent exacerbations, paroxysmal atrial fibrillation, and a history of aortic valve replacement eight years ago for severe aortic stenosis. She is currently taking rivaroxaban 10 mg orally daily, bisoprolol 2.5 mg orally daily, aspirin 100 mg orally daily, and furosemide 40 mg orally daily. The patient presented with symptoms of fever, dry cough, diarrhea, and bilateral flank pain. Upon presentation, the patient was clinically stable with a low-grade fever. Physical exams revealed regular heart sounds with an aortic click, bilateral crackles on lung auscultation without lower limb edema, no costovertebral angle tenderness, and a soft, non-tender abdomen. Laboratory tests were unremarkable except for a creatinine level of 2.6 mg/dl, indicating a glomerular filtration rate of 28 ml/min according to the Cockcroft-Gault formula. The electrocardiogram was normal; the anteroposterior chest X-ray (Figure 1) showed cardiomegaly, clear lungs, hilar prominence, and sternal wires. A CT scan of the abdomen/pelvis was normal. The infectious disease team considered her illness to be viral. Due to the patient's cardiac history, the cardiology team was consulted. During the medical history discussion, the patient mentioned having had a mechanical valve replacement, but her son denied it, reporting a biological valve replacement instead. The patient revealed she had been on acenocoumarol post-surgery but stopped it 18 months ago during the ongoing economic crisis in Lebanon and the subsequent unavailability of acenocoumarol due to the healthcare system collapse; she started taking a generic version of rivaroxaban 10 mg, which is less expensive than the brand name, without consulting her cardiologist and was being followed up by her general practitioner who likely provided her access to this drug at the dose of 10mg. Despite being less expensive, acenocoumarol was unavailable in Lebanon during the peak of the economic crisis and the collapse of the healthcare system because of high demand and the absence of any other form of anti-vitamin K. This issue of drug availability was later addressed by interventions from the Ministry of Health and other non-governmental agencies, through specific protocols providing medications to eligible patients.

It is notable that our patient was unaware of the availability of acenocoumarol through these governmental and non-governmental channels, as she had not consulted her cardiologist and had only been followed up by a general practitioner during this period.

**Figure 2:**
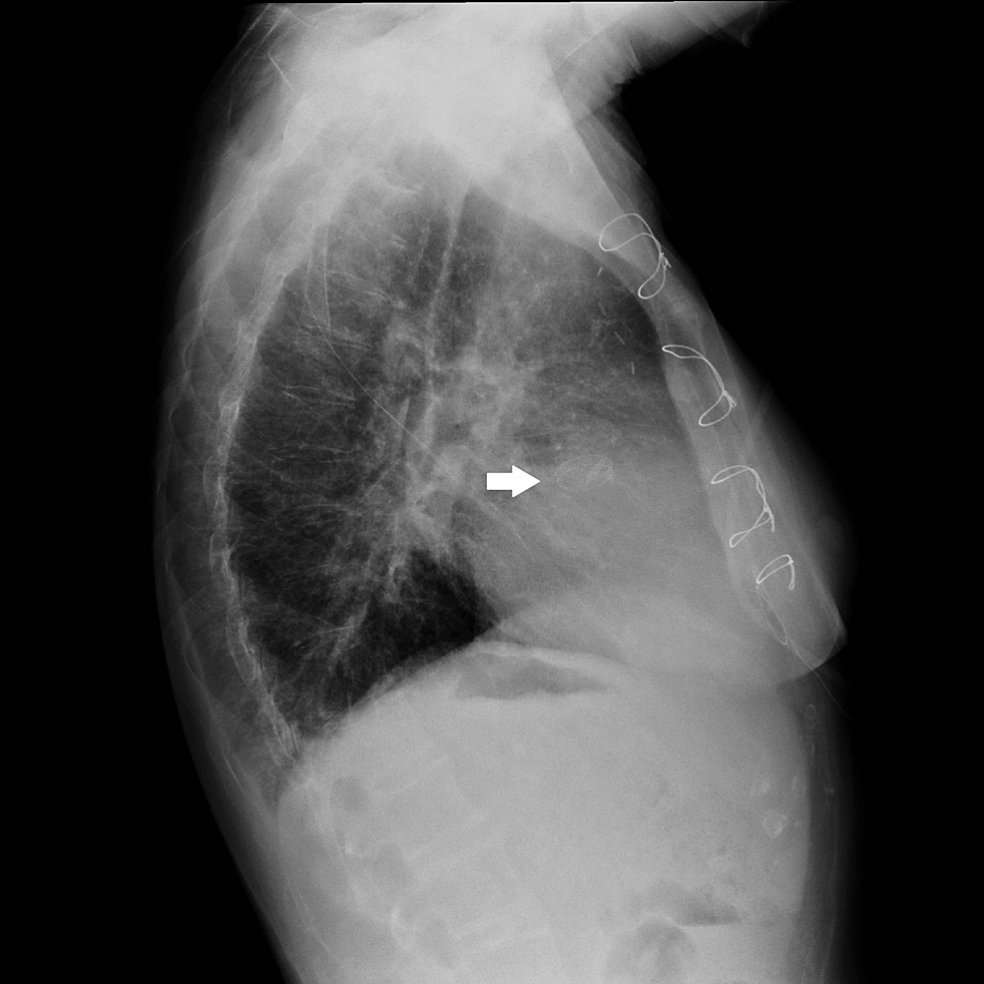
Lateral chest X-ray showing sternal wires, clear lungs, and a mechanical prosthesis projecting over the aortic valve position (indicated by a white arrow).

Further investigations were conducted, including a lateral chest X-ray, which indicated a possible mechanical aortic valve prosthesis (Figure 2).

**Figure 3:**
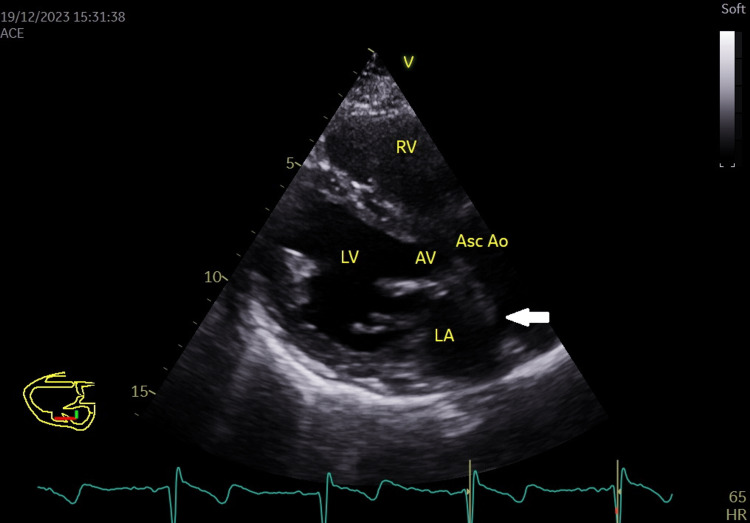
Parasternal long axis view from the left parasternal window showing the aortic mechanical valve with its reverberation (white arrow) in the ascending aorta and left atrium. Asc Ao: Ascending aorta; LA: Left atrium; LV: Left ventricle; RA: Right atrium; RV: Right ventricle.

**Figure 4:**
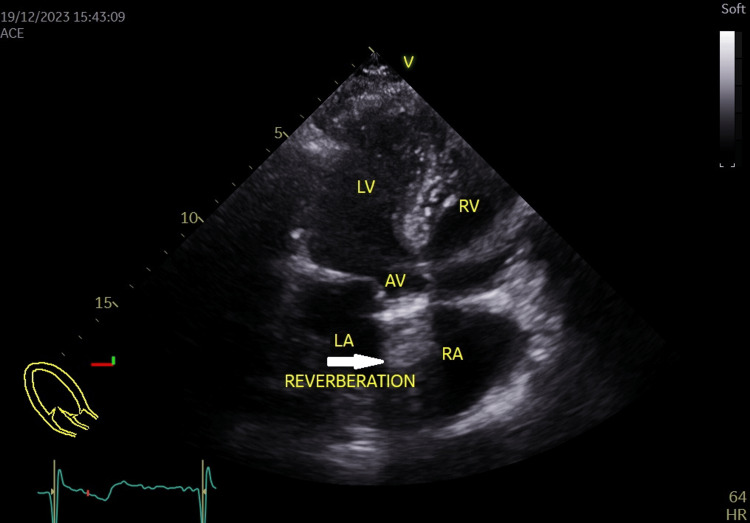
Apical 5 chambers view showing the aortic mechanical valve with its reverberation (white arrow) in the ascending aorta. AV: Aortic valve; LA: Left atrium; LV: Left ventricle; RA: Right atrium; RV: Right ventricle.

**Figure 5:**
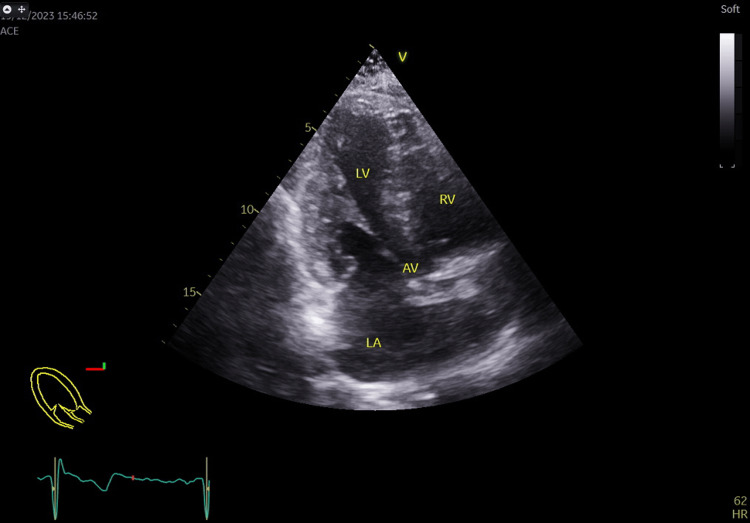
Apical long axis view showing the mechanical aortic valve with its reverberation in the ascending aorta. LA: Left atrium; LV: Left ventricle; RA: Right atrium; RV: Right ventricle.

**Figure 6:**
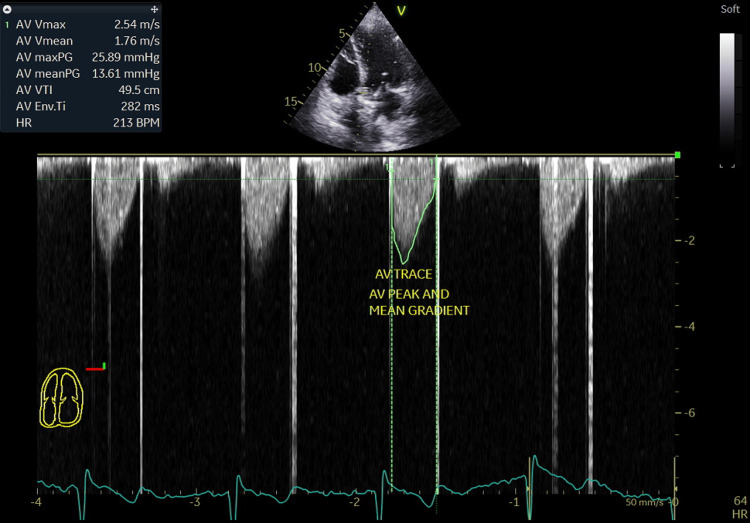
Aortic mechanical prosthetic valve peak systolic gradient recorded at 25.8 mmHg and mean systolic gradient at 13.6 mm Hg at a heart rate of 64 beats/minutes from the apical 5 chambers view. AV TRACE: Aortic valve trace; AV peak and mean gradient: Aortic valve prosthesis peak and mean pressure gradients.

**Figure 7:**
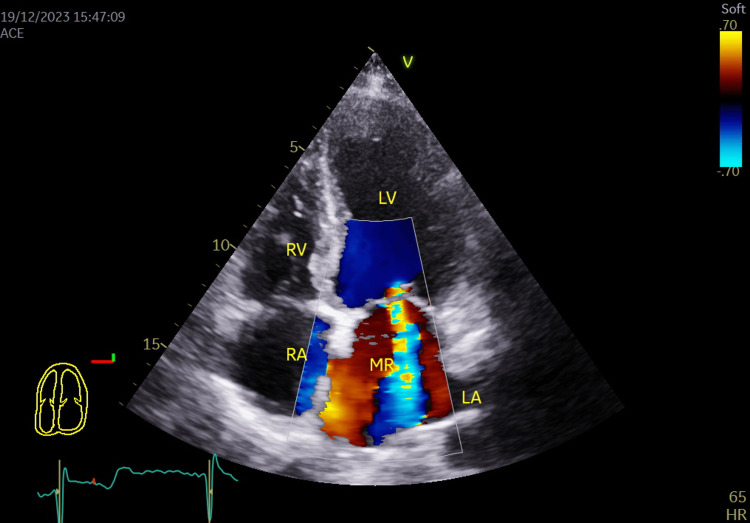
Color flow coding showing grade III mitral regurgitation in apical 4 chambers view. LA: Left atrium; LV: Left ventricle; RA: Right atrium; RV: Right ventricle; MR: Mitral regurgitation

**Figure 8:**
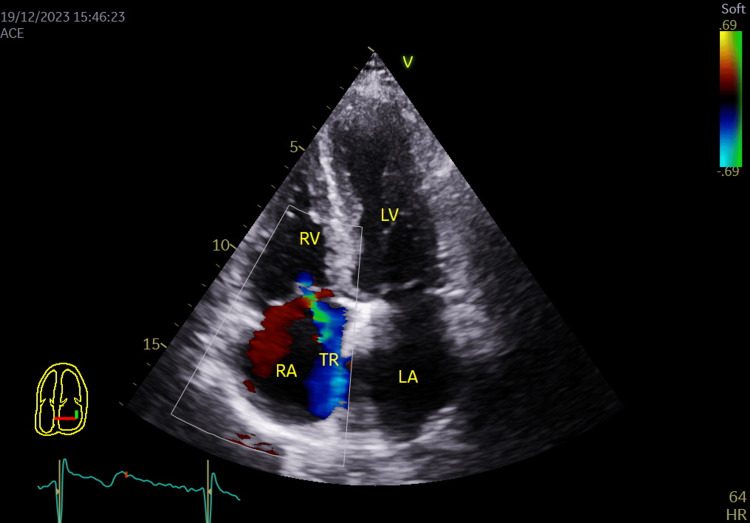
Color flow coding showing grade II tricuspid regurgitation in apical 4 chambers view. LA: Left atrium; LV: Left ventricle; RA: Right atrium; RV: Right ventricle; TR: Tricuspid regurgitation.

**Figure 9:**
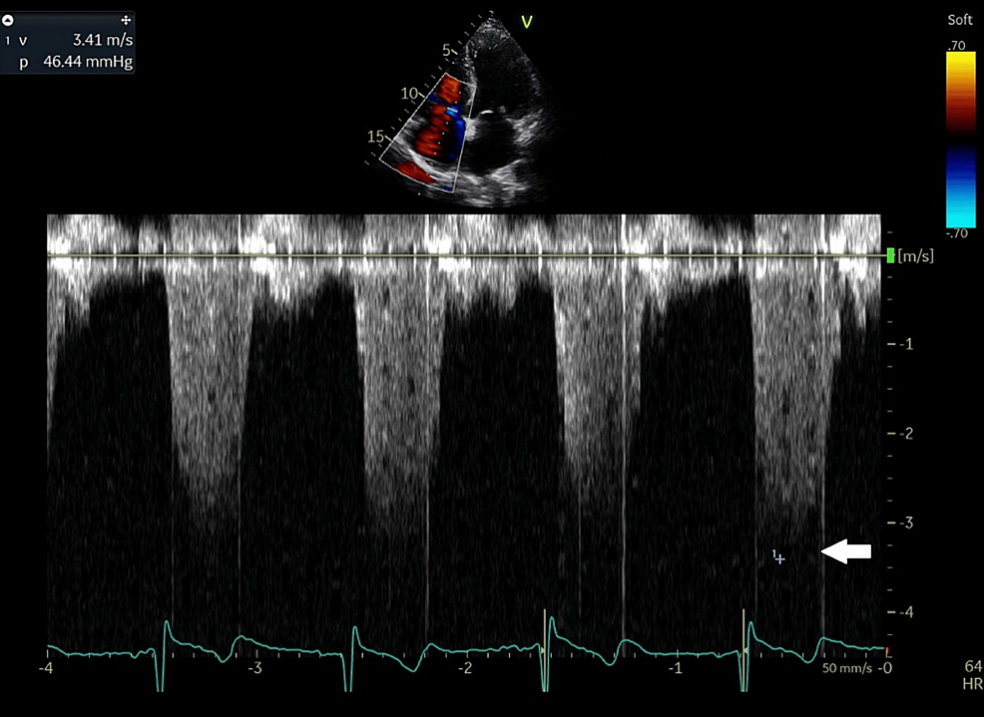
Tricuspid regurgitation jet velocity, recorded by CW Doppler in the apical 4-chamber view, was 3.41 m/s (indicated by a white arrow), allowing for the calculation of systolic pulmonary artery pressure at 51 mmHg. m/sec: meter/second; CW: Continuous wave.

**Figure 10:**
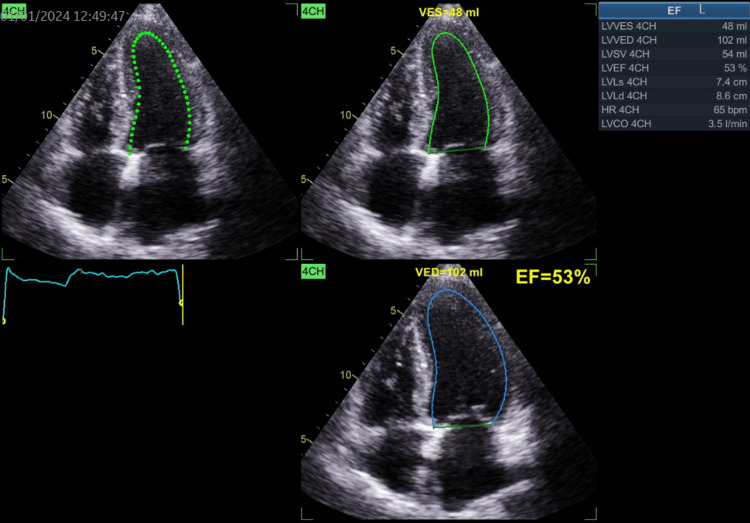
Left ventricular ejection fraction recorded at 53% from the apical four chambers view. EF: Ejection fraction; VED: Volume at end diastole; VES: Volume at end systole.

Subsequent echocardiography confirmed the presence of a normally functioning mechanical aortic valve prosthesis with a peak systolic gradient of 25.8 mmHg and a mean systolic gradient of 13.6 mmHg at a heart rate of 64 beats per minute (Figures 3-6), Grade III mitral regurgitation (Figure 7), Grade II tricuspid regurgitation (Figure 8) with a calculated systolic pulmonary artery pressure of 51 mmHg by continuous wave (CW) Doppler (Figure 9); the inferior vena cava was normal in size and collapsed > 50% with inspiration, allowing estimation of right atrial pressure at 5 mmHg. Left ventricular ejection fraction (LVEF) was preserved at 53% with hypokinesia of the inferior segment (Figure 10).

Video 1 shows the mechanical aortic valve from the apical long-axis view during echocardiography.

**Video 1:** Apical long axis view showing the mechanical aortic valve prosthesis. Source: Author Hasan Kazma.

**Figure 11:**
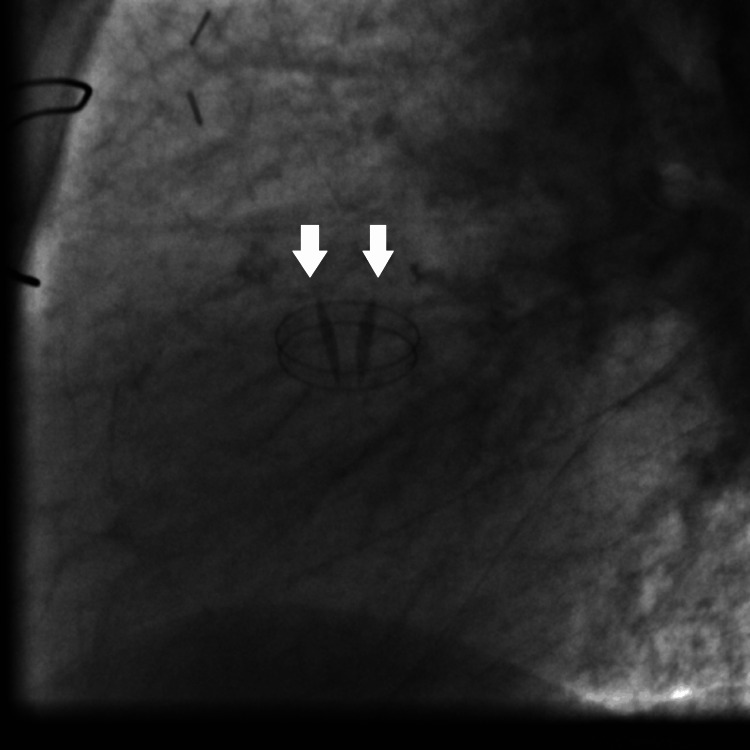
Fluoroscopy showing the mechanical aortic valve prosthesis leaflets in open position (white arrows).

**Figure 12:**
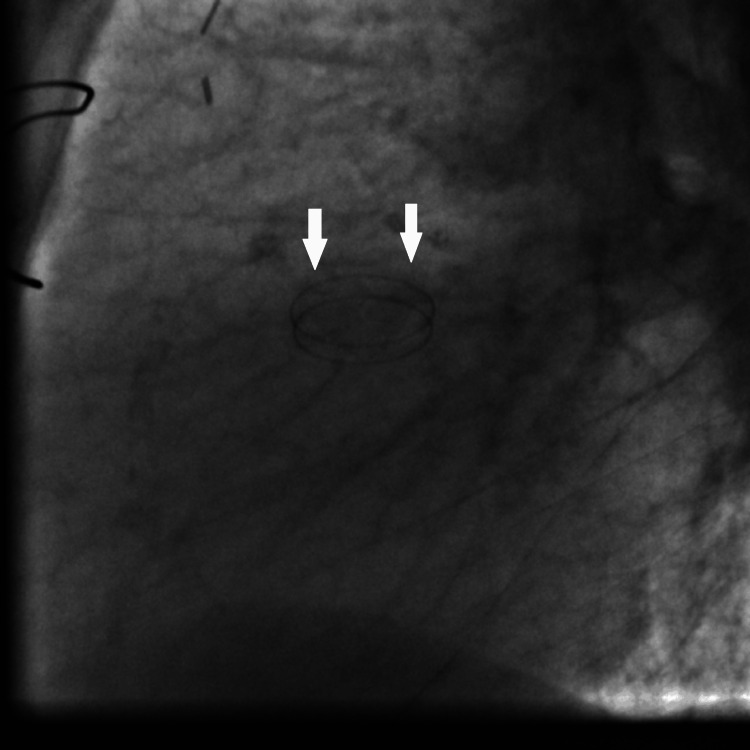
Fluoroscopy showing the mechanical aortic valve prosthesis leaflets in closed position (white arrows).

Fluoroscopy confirmed the presence of a well-functioning mechanical aortic valve prosthesis and demonstrated normal leaflet openings (Figure 11) and closing positions (Figure 12).

Video 2 shows the fluoroscopy of the mechanical aortic valve prosthesis functioning normally.

**Video 2:** Fluoroscopy of the mechanical aortic valve prosthesis showing normal opening and closing of the mechanical valve discs. Source: Author Hasan Kazma.

Simultaneously, we requested the old operative report from the hospital where the valve replacement was performed. It documented the placement of a 19 mm diameter mechanical aortic valve, identified as the "On-X" valve. The indication for aortic valve replacement was severe aortic stenosis, and coronary angiography showed non-occlusive coronary artery disease at that time. The diagnosis was ischemic cardiomyopathy with Grade III mitral regurgitation and preserved ejection fraction with pulmonary hypertension; the aortic mechanical valve prosthesis was functioning normally despite an 18-month period of anticoagulation with a non-indicated anticoagulant (rivaroxaban).

On the same day, the patient was started on acenocoumarol 3 mg daily with heparin bridging (24,000 units over 24 hours), monitored with partial thromboplastin time (PTT) and INR; PTT was adjusted to 2.5 times control. When the INR reached between 2.5 and 3.0, intravenous heparin was discontinued; bisoprolol 2.5 mg orally daily, aspirin 100 mg orally daily, and furosemide 40 mg orally daily were continued. Aspirin was restarted because of the combined coronary artery disease (segmental wall motion abnormalities with inferior segment hypokinesia and ischemic mitral regurgitation observed on echocardiography) and since we could not ascertain the timing when the patient may have developed her acute coronary syndrome leading to these findings. The patient was also started on atorvastatin 40 mg daily for secondary prevention. The patient was informed about her condition, educated on the importance of medication compliance and INR monitoring, and strongly advised never to change her treatment without consulting her cardiologist. The presence of ischemic cardiomyopathy and the importance of treating the associated significant mitral regurgitation (surgically or percutaneously) for symptom control and reduction in morbidity and mortality were discussed with the patient, and she decided to re-consult her cardiac surgeon for further therapy.

## Discussion

Aortic stenosis is the most prevalent valvular heart disease, particularly affecting the elderly population. Approximately 10% of individuals reach the age of 80 with this condition, manifesting symptoms related to left ventricular outflow obstruction, such as angina, syncope, and left ventricular heart failure [6,7]. Symptomatic aortic stenosis is closely linked to a poor prognosis and a high mortality rate if left untreated [8].

Aortic valve stenosis may be congenital, seen in 2% of the population with a bicuspid aortic valve, which exhibits a male predominance. Alternatively, it can be acquired, primarily caused by aging and the degenerative pattern of the valve. This explains why individuals with a bicuspid aortic valve develop stenosis at a younger age, typically before the age of 70, while those with a normal tricuspid aortic valve tend to develop stenosis after the age of 70 [9]. Other contributing factors include rheumatic heart disease due to chronic inflammation, dyslipidemia, and chronic kidney disease [10].

Transthoracic echocardiography is the primary imaging modality for evaluating aortic valve stenosis, providing insights into the cause and severity of the stenosis. Severe aortic stenosis is generally defined by a valve area ≤ 1 cm² and either a high peak velocity (> 4 m/s) or a high mean pressure gradient (> 40 mmHg) [9]. Additional assessment modalities include ECG, cardiac CT angiogram, and cardiac magnetic resonance [11].

For severe stenosis, the only effective treatment is valve replacement, coupled with risk factor modifications, such as addressing hypertension and dyslipidemia through antihypertensive and lipid-lowering therapy [9].

Surgical aortic valve replacement, utilizing either mechanical or biological prostheses, or transcatheter aortic valve replacement with a biological prosthesis, is recommended for patients with symptomatic severe aortic stenosis. It is also indicated for those with asymptomatic severe stenosis and a left ventricular ejection fraction less than 50%, or asymptomatic severe stenosis in individuals undergoing other cardiac surgery. In certain cases, it may be reasonable for patients with asymptomatic severe stenosis who develop symptoms, experience a decrease in blood pressure > 10 mmHg with exercise, exhibit very severe stenosis (defined by a transvalvular velocity > 5 m/s), have high serum brain natriuretic peptide (BNP > 3x normal), or show rapidly progressive stenosis [3].

Mechanical valve prostheses are highly durable but thrombogenic, with thrombosis incidences ranging from 0.1% to 5.7% per year, necessitating lifetime anticoagulation. In contrast, bioprosthetic valves are less durable but carry minimal thrombogenic risk and do not require anticoagulation. Therefore, mechanical valves are typically recommended for individuals younger than 50 years or those already on anticoagulation [3,4].

According to the American College of Cardiology/American Heart Association (ACC/AHA) guidelines, patients with mechanical valve prostheses should undergo anticoagulation using vitamin K antagonists, such as warfarin or acenocoumarol, with the target INR dependent on the valve type, location, and thrombosis risk. Currently, there is no evident role for DOACs in this context; studies have shown that the use of dabigatran in patients with mechanical valves is associated with an increased risk of stroke and bleeding. Thus, there is sufficient clinical trial data on DOACs in patients with mechanical heart valves to recommend withholding their usage in this patient population [4,5].

Back to our case, the patient had undergone mechanical aortic valve replacement for aortic stenosis and had been receiving rivaroxaban for 18 months without consulting her cardiologist, but was followed up by her general practitioner due to the unavailability of acenocoumarol during the economic crisis and collapse of the healthcare system in Lebanon. Remarkably, despite this deviation from conventional treatment and the inherent risks associated with mechanical valve prostheses, the patient remained free of complications such as valve thrombosis or dysfunction for 18 months. This anecdotal observation diverges from the higher complication rates reported in clinical trials for patients with mechanical valve prostheses receiving DOACs compared to those receiving antivitamin K therapy. However, it's important to note that major cardiology societies still contraindicate DOAC use in this patient population [4,5]. The fact that no complications have arisen until now does not guarantee that the patient will remain complication-free if she continues to take rivaroxaban. Our case report highlights the critical importance of accurate diagnosis and appropriate anticoagulation therapy, particularly in the context of valve replacement, especially with mechanical valve prostheses. This significance is especially relevant for primary care physicians, including general practitioners and family medicine physicians, who may oversee the ongoing care of such patients. This case exemplifies the need for vigilance as the patient had undergone mechanical aortic valve replacement, had been taking rivaroxaban, and was followed up by her general practitioner. Thus, the primary aim of this case report is to provide vital education to primary care physicians, emphasizing the essential role they play in the comprehensive management of patients with valve replacements and the importance of collaboration with specialists to ensure optimal patient care. In times of economic crisis and healthcare system collapse, as seen in Lebanon, it becomes imperative for primary care physicians to possess comprehensive knowledge about mechanical heart valves, educate patients about their condition and the potential risks associated with DOACs, and assist patients in securing critical medications through governmental and non-governmental channels in the event of healthcare system breakdowns.

"Upon clinical interview and physical examination, suspicion arose regarding the type of valve prosthesis, ultimately leading to confirmation through imaging studies that the patient indeed had a mechanical valve. Given this discrepancy and the recent interventions by governmental and non-governmental agencies to provide drugs for eligible patients through specific protocols, the medical team swiftly transitioned the patient to acenocoumarol, optimizing her treatment regimen based on the identified valve type and evidence-based medicine. Notably, the patient was unaware of the availability of acenocoumarol through these agencies, as she had not consulted her cardiologist and had been followed up only by a general practitioner during this period. We took immediate steps to educate the patient on the specific indications for acenocoumarol, emphasizing its importance in preventing blood clots around the mechanical valve. Additionally, we discussed the contraindications of rivaroxaban in this context. To ensure ongoing safety and efficacy, we empowered the patient with information on the necessity of regular INR monitoring, underscoring the importance of maintaining a therapeutic range for effective anticoagulation. This intervention not only corrected a potentially life-threatening situation but also equipped the patient with the knowledge needed to actively participate in her ongoing care and well-being. In fact, the entire ‘journey’ of imaging provided our patient with the strong education she needed to have an active participation in her medical management and well-being. Concerning her ischemic cardiomyopathy and severe mitral regurgitation, the patient was referred back to her cardiac surgeon for further therapy: coronary angiography and subsequent percutaneous or surgical therapy for both coronary artery disease and severe mitral regurgitation."

## Conclusions

Despite the absence of complications during the patient's use of a DOAC, it is crucial to recognize that these medications are contraindicated for individuals with mechanical cardiac valves. However, as in our case, having no complications up to now does not guarantee that the patient will not develop complications later if she continues to take rivaroxaban. This situation emphasizes the need for an accurate assessment of the valve type through a detailed history, physical exam, review of available medical records, and imaging studies, with appropriate treatment tailored to the patient's valve type. This issue is especially important when patients are followed by their primary care physician. Primary care physicians, who often oversee the care of such patients, play a critical role in ensuring optimal management. Moreover, it falls within their purview to assist patients in securing critical medications (like acenocoumarol) through governmental and non-governmental channels in the event of a healthcare system breakdown, as happened in Lebanon. This case also underscores the importance of patient education and collaboration among healthcare providers. Ultimately, it serves as a reminder of the complexities involved in managing valvular heart disease.
